# Genome Sequence of the Sulfate-Reducing Thermophilic Bacterium Thermodesulfovibrio yellowstonii Strain DSM 11347^T^ (Phylum *Nitrospirae*)

**DOI:** 10.1128/genomeA.01489-14

**Published:** 2015-01-29

**Authors:** Srijak Bhatnagar, Jonathan H. Badger, Ramana Madupu, Hoda M. Khouri, Elizabeth M. O’Connor, Frank T. Robb, Naomi L. Ward, Jonathan A. Eisen

**Affiliations:** a Microbiology Graduate Group, University of California Davis, Davis, California, USA; b J. Craig Venter Institute, La Jolla, California, USA; c J. Craig Venter Institute, Rockville, Maryland, USA; d Institute of Marine and Environmental Technology, and Department of Microbiology and Immunology, University of Maryland, Baltimore, Maryland, USA; e Department of Molecular Biology, University of Wyoming, Laramie, Wyoming, USA; f Department of Evolution and Ecology, UC Davis Genome Center, Department of Medical Microbiology and Immunology, University of California Davis, Davis, California, USA

## Abstract

Here, we present the complete 2,003,803-bp genome of a sulfate-reducing thermophilic bacterium, Thermodesulfovibrio yellowstonii strain DSM 11347^T^.

## GENOME ANNOUNCEMENT

Thermodesulfovibrio yellowstonii is a sulfate-reducing, strictly anaerobic bacterium first isolated from the Sedge Bay of Yellowstone Lake in Wyoming, USA. It is a Gram-negative bacterium with curved rod-shaped cells averaging 1.5 µm in length and 0.3 µm in width. It is a motile organism, propelled by a single polar flagellum. T. yellowstonii can use sulfate, thiosulfate, and sulfite as terminal electron acceptors (1). It grows between the temperature range of 40°C and 70°C, with optimal growth at 65°C. The genome of T. yellowstonii was sequenced as part of an “Assembling the Tree of Life” project at the Institute for Genomic Research (TIGR). At the time that the project started (2002), there were no genomes available from the phylum *Nitrospirae*, of which T. yellowstonii is a member.

The type strain of T. yellowstonii (DSM 11347^T^) was obtained from the Leibniz Institute DSMZ-German Collection of Microorganisms and Cell Cultures and grown anaerobically at 65°C using DSMZ medium 749. DNA was obtained by solubilizing cells with *N*-lauryl sulfate and sodium dodecyl sulfate, followed by incubation with proteinase K. The lysate was extracted with tris-EDTA-saturated phenol and chloroform/isoamyl alcohol and was precipitated from the aqueous phase with 95% ethanol. It was resolubilized, incubated with DNase-free RNase and further purified by cesium-chloride gradient centrifugation, and visualized using 365 nm UV light (2). Pulse-field gel electrophoresis was used to confirm the size and uniformity of the DNA preparation. Genome sequencing was performed in the following way: small (2 to 3 kb), medium (4 to 5 kb), and large (8 to 10 kb) insert libraries were made and Sanger sequenced, and assemblies were generated as previously described (3–5); assemblies were edited and gaps were closed by clone walking and targeted PCR and sequencing. Finishing was completed by generating additional coverage in low-coverage regions, verification of repeats, and resolution of ambiguities (6). The final assembly had ~9× coverage for the 2,003,803-bp genome and a GC content of 34.13%.

The origin of replication was identified using GC skew and colocalization of origin-associated genes (7). All the universal single-copy bacterial marker genes (8) were found in the sequenced genome using Phylosift (9). The genome was annotated as previously described (10). Of the 2,084 putative genes that were identified in the genome, 2,029 were putative protein-coding sequences (CDS) and 54 were putative noncoding RNA genes (3 noncoding RNAs, 3 rRNAs, 1 transfer-messenger RNA, and 47 tRNAs). Additionally, CRISPRFinder (11) identified five CRISPR repeats in the genome.

### Nucleotide sequence accession numbers.

The genome sequence has been deposited at GenBank under the accession number CP001147 and has been curated by NCBI staff under the accession number NC_011296. The version NC_011296.1 described in this paper was last modified on 20 March 2014.
